# Quantifying Reproducibility in Computational Biology: The Case of the Tuberculosis Drugome

**DOI:** 10.1371/journal.pone.0080278

**Published:** 2013-11-27

**Authors:** Daniel Garijo, Sarah Kinnings, Li Xie, Lei Xie, Yinliang Zhang, Philip E. Bourne, Yolanda Gil

**Affiliations:** 1 Ontology Engineering Group, Facultad de Informática, Universidad Politécnica de Madrid, Madrid, Spain; 2 Department of Chemistry and Biochemistry, University of California San Diego, La Jolla, California, United States of America; 3 Skaggs School of Pharmacy and Pharmaceutical Sciences, University of California San Diego, La Jolla, California, United States of America; 4 Department of Computer Science, Hunter College, The City University of New York, New York, New York, United States of America; 5 School of Life Sciences, University of Science and Technology of China, Hefei, Anhui, China; 6 Information Sciences Institute and Department of Computer Science, University of Southern California, Los Angeles, California, United States of America; The Centre for Research and Technology, Hellas, Greece

## Abstract

How easy is it to reproduce the results found in a typical computational biology paper? Either through experience or intuition the reader will already know that the answer is with difficulty or not at all. In this paper we attempt to quantify this difficulty by reproducing a previously published paper for different classes of users (ranging from users with little expertise to domain experts) and suggest ways in which the situation might be improved. Quantification is achieved by estimating the time required to reproduce each of the steps in the method described in the original paper and make them part of an explicit workflow that reproduces the original results. Reproducing the method took several months of effort, and required using new versions and new software that posed challenges to reconstructing and validating the results. The quantification leads to “reproducibility maps” that reveal that novice researchers would only be able to reproduce a few of the steps in the method, and that only expert researchers with advance knowledge of the domain would be able to reproduce the method in its entirety. The workflow itself is published as an online resource together with supporting software and data. The paper concludes with a brief discussion of the complexities of requiring reproducibility in terms of cost versus benefit, and a desiderata with our observations and guidelines for improving reproducibility. This has implications not only in reproducing the work of others from published papers, but reproducing work from one’s own laboratory.

## Introduction

Computation is now an integral part of the biological sciences either applied as a technique or as a science in its own right - bioinformatics. As a technique, software becomes an instrument to analyze data and uncover new biological insights. By reading the published article describing these insights, another researcher hopes to understand what computations were carried out, replicate the software apparatus originally used and reproduce the experiment. This is rarely the case without significant effort, and sometimes impossible without asking the original authors. In short, reproducibility in computational biology is aspired to, but rarely achieved. This is unfortunate since the quantitative nature of the science makes reproducibility more obtainable than in cases where experiments are qualitative and hard to describe explicitly.

An intriguing possibility where potential quantification exists is to extend articles through the inclusion of scientific workflows that represent computations carried out to obtain the published results, thereby capturing data analysis methods explicitly [1]. This would make scientific results more reproducible because articles would have not only a textual description of the computational process described in the article but also a workflow that, as a computational artifact, could be analyzed and re-run automatically. Consequently, workflows can make scientists more productive because they capture complex methods in an easy to use accessible manner [2]–[3].

The goal of this article is, by applying a workflow to an existing computational analysis [4], to describe and quantify the effort involved in reproducing the published computational method and to articulate guidelines for authors that would facilitate reproducibility and reuse. Quantification is achieved by assigning a reproducibility score that exposes the cost of omitting important information from the published paper that then caused problems in creating the workflow. Beyond this no case is made for the value of workflows which is well described elsewhere [3].

### Related Work

As stated, scientific articles describe computational methods informally, as the computational aspects of the method may not be the main focus of the article. We acknowledge that in computer science the method may be described formally and any limitations, it could be argued, reside with the editors and reviewers. However, in the domain of computational biology, which is the focus here, we believe methods to be, for the most part, described informally as formalizations are not typically favored by authors or enforced by reviewers.

Computational methods are often complex and hard to explain in textual form with the given space limitations of many articles. As a result, reproducing methods often requires significant effort from others to reproduce and reuse. Studies have shown that reproducibility is not achievable from the article itself, even when datasets are published [5]–[7]. The reproducibility process can be so costly that it has been referred to as “forensic” research [8]. Lack of reproducibility also affects the review process and as a result retractions of publications occur more often than is desirable [9]. A recent editorial proposed tracking the “retraction index” of scientific journals to indicate the proportion of published articles that are later found problematic [10]. Publishers themselves are asking the community to end “black box” science that cannot be easily reproduced [11]. Pharmaceutical companies report abandoning efforts to reproduce research that seemed initially promising and worth investigating after substantial investments [12].

Computational reproducibility is a relatively modern concept. The Stanford Exploration Project led by Jon Claerbout published an electronic book containing a dissertation and other articles from their geosciences lab [13]. Papers are accompanied by zipped files with the software that could be used to reproduce the results, and a methodology was developed to create and manage all these objects that continue today with the Madagascar software [14]. Advocates of reproducibility have sprung up over the years in many disciplines, from signal processing [15] to psychology [16]. Organized community efforts include reproducibility tracks at conferences [17]–[19], reproducibility editors in journals [20], and numerous community workshops and forums (e.g., [21], [22]). Active research in this area is addressing a range of topics including copyright [23], privacy [24], social [25] and validation issues [26].

Scientific publications could be extended so that they incorporate computational workflows, as many already include data [1]. Without access to the source codes for the papers, reproducibility has been shown elusive [7]. This would make scientific results more easily reproducible because articles would have not just a textual description of the computational process used but also a workflow that, as a computational artifact, could be inspected and automatically re-executed. Some systems exist that augment publications with scripts or workflows, such as Weaver for Latex [27]–[28] and GenePattern for MS Word [29]. Many scientific workflow systems now include the ability to publish provenance records [30]–[31]. The Open Provenance Model was developed by the scientific workflow community and is extensively used for this purpose [32]. Here we make a contribution to the on-going discussion of reproducibility by attempting to quantify what reproducibility implies.

## Methods and Analysis

### Quantifying Reproducibility

We focus on an article that describes a method that lends itself to workflow representation, since others can, in principle, use the same exact procedures [4]. The article describes a computational pipeline that, as applied, maps all putative FDA and European drugs to possible protein receptors within a given proteome; Mycobacterium tuberculosis (TB) in the paper under study. Mapping is limited to the accessible structural proteome of experimental structures and high quality homology models. Mapping is performed using a binding site comparison algorithm which compares the binding site of the drug bound to a primary protein receptor to potential binding sites found on every available protein in a given proteome. Docking of the drug to the off-target protein is used to further validate the predicted binding. The study uses data from the RCSB Protein Data Bank (PDB [33]) and Modbase [34]. The resultant “drugome” established multiple receptors to which a given drug can bind and multiple drugs that could bind to a given receptor. As such it is a putative map of possible drug repositioning strategies in treating a given condition caused by a pathogen. Although the article focuses on Mycobacterium tuberculosis (TB), according to the article’s abstract:

“… the methodology may be applied to other pathogens of interest with results improving as more of their structural proteomes are determined through the continued efforts of structural biology/genomics.”

That is, the methodology is likely to be repeated for other organisms and/or repeated in the same organism as more drugs become available and/or more of the structural proteome becomes available. The original work did not use a workflow system; instead the computational steps were run separately and manually. The original work was done over a period of two years, with different authors having different degrees of participation in the design and the programming aspects of the study. There is a TB Drugome project site where many details about the work can be found [35].

The original article was used to challenge participants at the first *Beyond the PDF* workshop [21]. The workshop attracted participants interested in bettering the communication and comprehension of science. The challenge was to apply the tools they had developed to illustrate their value on a given piece of science to which, as far as possible, all lab notes, raw data, software, drafts of the paper etc. where made available. The work described here is one outcome of these efforts and is aimed at addressing the questions: What can we gain from the process of workflow creation and what does it tell us about reproducibility?

The rest of this paper describes our attempt to answer these questions. Many details of the analysis and how progress was made in reproducing the method are available on the project site [36]. Also Supplement S1 includes a more detailed analysis and the thought processes that occurred.

### Methodology

The workflow was reproduced as a joint effort between computer scientists and the original authors of the article. Although some of the authors of the paper had moved to other research groups (notably Kinnings, its first author), they were still available to answer questions and provide software scripts and data as needed.

We present a detailed analysis of the issues that came up in reproducing three major parts of the methods section in the original paper. These three parts were originally fully automated. Other steps of the method, notably the initial steps to obtain the data and the final steps for visualization and presentation, were manually done and not considered as part of the workflow presented here.

We describe how each of the three method subsections was implemented as a workflow. Each computational step corresponds to an execution of an existing tool or a script written by the paper authors. We were able to recreate the workflow in the Wings workflow system [37]–[39] to make sure it was executable and reproduced the original results reported in the paper. Hence, the workflow explicitly represents the method that the authors meant to convey in the original text, that is, the process by which software and data are used to achieve the published result.

Based on this explicit computational workflow, we present an analysis of the reproducibility of each subsection. We considered reproducibility by researchers of four types:

1. **REP-AUTHOR**, is a researcher who did the original work and who may need to reproduce the method to update or extend the results published. It is assumed that the authors have enough backup materials to answer any questions that arise in reconstructing the method. In practice, some authors may be students that move away from the lab and their materials and notes may or may not be available, confounding reproducibility [40].
2. **REP-EXPERT** is a researcher familiar with the research area. These researchers could reproduce the method even if the methods section of the paper is incomplete and ambiguous. They can use their knowledge of the domain, the software tools and the process to make very complex inferences from the text and reconstruct the method. However, there may be some non-trivial inferences that require significant effort.
3. **REP-NOVICE** is a researcher with basic bioinformatics expertise. They may be asked to use the method with new data, but are only able to make limited inferences based on analyzing the text and software tools. For them reproducibility can be very costly since it may involve a lot of trial and error, or perhaps additional research. In some cases reproducibility may become impossible.
4. **REP-MINIMAL** is a researcher with no expertise in bioinformatics. They need some programming skills to assemble the software necessary to run the different steps of the method. They represent researchers from other areas of science with minimal knowledge about biology, students, and even entrepreneurial citizen scientists (e.g., [41]). Unless the steps of the method are explicitly stated, they would not be able to reproduce the results.

In our work, we did not ask experts to reproduce the method, so we only have three categories of researcher rather than four. We used the following approach:

- **REP-MINIMAL** - The computer scientists in the team read the article and formulated the initial workflows. They have minimal background knowledge in biology.
- **REP-NOVICE** - The computer scientists subsequently consulted the documentation on the software tools mentioned in the article to try to infer how the data were being processed by each of the steps of the method. Based on this, they refined their initial workflows.
- **REP-AUTHOR** - Lastly the computer scientists approached the original paper authors to ask specific questions, resolve execution failures and errors and consult concerning the validity of the results for each step. They created the final workflow based on these conversations with the authors.

We analyzed each of the workflow steps in terms of: whether the existence of the step itself was clear to the reproducers, whether the software that was used to run the step was clear to the reproducers, and whether their inputs and outputs were clear. For example, the existence of a step to compare ligand binding sites is mentioned in the text of the original paper, and the fact that it was carried out using the SMAP software [42] is also explicit in the text, so those would be things that the REP-MINIMAL reproducers were able to figure out. The use of a p-value as an input was not mentioned in the text and cannot be easily inferred unless the researcher reproducing the method becomes familiar with the software, so REP-NOVICE reproducers were able to figure out this parameter.

For this analysis, we assigned a reproducibility score to each aspect of the workflow for each of these reproducer categories. A score of 1 in a category means that, in our assessment, a prototypical researcher of that category would be able to figure out the item. A score of 0 means that they would not be likely to figure it out without help from experts.

Based on these scores, we designed a reproducibility map, where the reproducibility of each computational step was highlighted to determine how far each category of researcher could go in reproducing a given workflow fragment.

Finally, we report on the effort involved in creating the workflow, measured as the time spent on various aspects of the work involved in reproducing the method described in the original article.

### Conceptual Overview of the Method and Final Workflow

**Figure 1:**
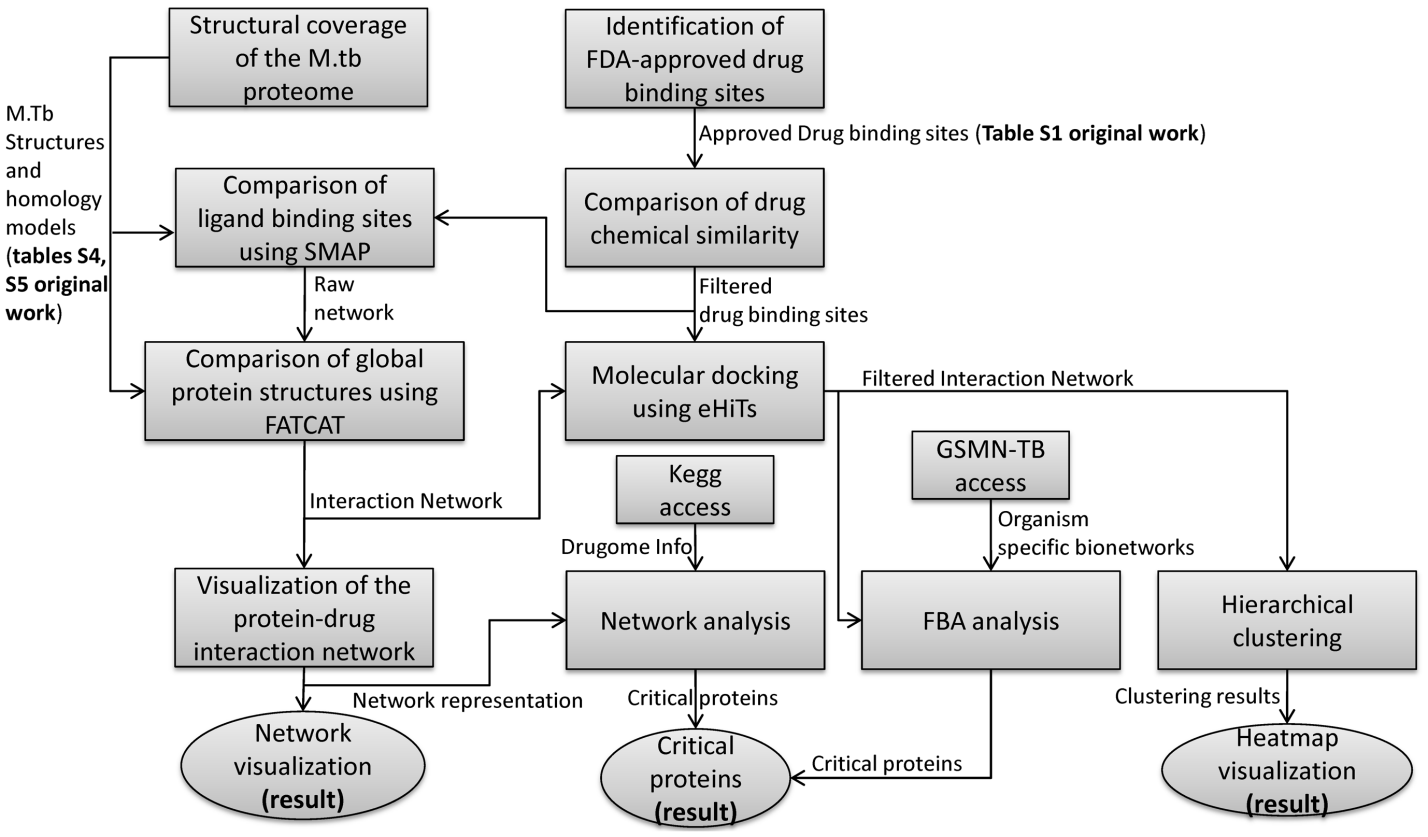
A high-level dataflow diagram of the TB drugome method.

An interesting result of our initial discussions of the method was a collaborative diagram that indicated each of the steps in the method and how data were generated and used by each step. This diagram, shown in Figure 1, makes the steps of the method more explicit and adds useful information to the text in the methods section. It also shows where the data in the tables of the article fit into the method.

In essence, the bulk of the results in the paper are obtained through three major steps:

1. *Comparison of ligand binding sites*, which compares the putative binding sites of solved protein structures and homology models (obtained from queries to the PDB and other sources) against the binding sites from protein structures where approved drugs are bound. This step used the SMAP software [42].
2. *Comparison of protein structures*, optimizing their alignment as well as reporting on the statistical significance of the structural similarity. This step used the FATCAT software [43] and is in essence a filtering step to remove structures which have overall global similarity and hence likely to be in the same protein family, since we are interested in similar binding sites found in otherwise dissimilar proteins.
3. *Molecular docking*, to predict the binding and affinity of the proteins and drug molecules. This step used the eHits software [44].

**Based on our experience, authors should be encouraged to publish such high-level flow diagrams as a normal part of the materials and methods section of a paper.** The diagrams provide a high level overview of the method, highlights major steps, and offer a roadmap for reproducibility.

**Figure 2:**
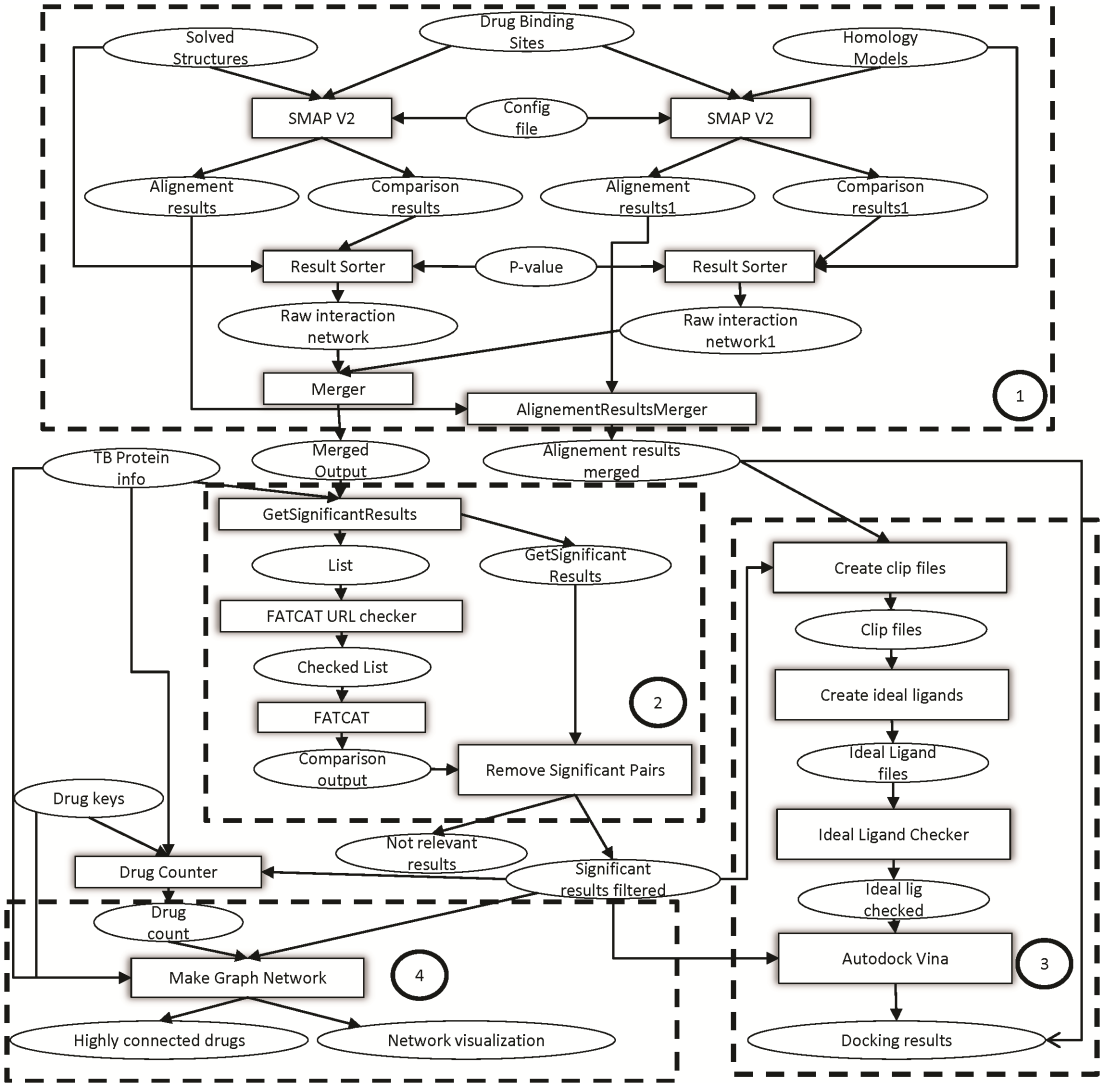
The reproduced TB Drugome workflow with the different subsections highlighted. (1) Comparison of ligand binding sites using SMAP; (2) protein structure comparison using FATCAT; (3) docking using Autodock Vina; and (4) graph network creation (visualization). We focus on the reproducibility of sections 1-3 here.

The final workflow with the four steps that reproduced the method is shown in Figure 2. We highlight the first three major subsections of the method. In order to validate the new results, we used the same inputs (drug binding sites, solved structures, and homology models) as in the original work. However, these inputs point to external data sources (like the PDB) where the data are stored. These third-party data sources had been updated, and therefore the workflow execution produced slightly different results than the results reported in the original article. A detailed comparison of the original results and the results of the new workflow is provided in Supplement S1.

### Reproducibility Analysis

We now analyze each of the subsections of the method as described in the original paper, discussing the difficulties encountered in reproducing the method, highlighting recommendations to improve reproducibility, and show reproducibility scores for each step of the final workflow. An extended analysis of each subsection of the method is available in Supplement S1, detailing the evolution of each sub-workflow in order to achieve the final result.

**Comparison of ligand binding sites.** The initial workflow design used a single step to compare the three items: the binding sites of experimental structures, the binding sites of the homology models, and the binding sites of the proteins to which drugs were bound. Examining the SMAP software and associated scripts revealed that comparison occurred in two steps: one to compare the experimental binding sites with the drug binding sites, and one to compare the homology model binding sites with the drug binding sites.

To clarify how the outputs of both SMAP invocations were combined, the authors provided the script that invoked the SMAP software. This revealed a new step for sorting the results. In addition, there was an additional step where the results below a given p-value were filtered out.

The SMAP software has several configuration parameters. Without the author’s configuration files, default values of the parameters were used not knowing if the workflow would produce questionable results. That is, it is not clear whether without the same parameter settings the original method would be reproduced and similar results would be obtained. For these reasons, the original configuration files were obtained from the authors. **This suggests that it would be good practice for authors to publish not just a description of the software used and the data used in the original experiment, but also the configuration files used.**

It also became clear that the data published as tables in the original article were not the direct input to the SMAP software, and some transformations would be required in order to use these data in the workflow. **We recommend that when data is published in formats that make it more readable, the actual data that is input for software to run also be made available.**

Another issue concerned the constant evolution of the software tools that are used for the method steps. In our case, the SMAP software had evolved since the publication of the original paper. As with many software tools used in biology, SMAP is an active research effort and its functionality continues to improve. When the workflow was reproduced there was a new version of SMAP that had the same basic functionality, but produced slightly different results. Under normal research circumstances, it is not critical that the workflow reproduce the exact execution results, but that the conclusions drawn from those results still hold. An interesting result would be if the workflow was run again with a newer more powerful tool and there were additional findings over and above the original publication. The same can be said for new and more comprehensive sources of input data. **The possibility of easily re-running and checking the method periodically with new versions of software tools and/or data that might lead to additional findings may entice researchers to keep their methods more readily reproducible.**

**Global comparison of protein structures.** Inspecting the scripts used by the authors revealed two steps for this subsection not mentioned in the original article. The first step generates a list of significant comparisons, which is used in the second step to remove significantly similar pairs of global structures from the FATCAT output. An expert in the domain would infer the need for these steps from the published article – only one structure from a set with similar global structures is needed to reach the appropriate conclusions. The article mentions the use of a threshold of 0.05, but this value did not appear in any parameter file. The FATCAT documentation mentions that 0.05 is a default value used to filter results, so this threshold did not have to be reflected in the workflow since it was fixed by the software – hard for a novice to know. Thus the workflow for this subsection could not be recreated just from the article alone, but required the scripts from the authors. Authors should be encouraged to publish any software and parameter files that were written by them and that became part of the method, because public domain software tools are only part of the software required to reproduce the method.

An important issue regarding reproducibility came up in this subsection of the workflow. Although the method was reproduced with all of the necessary steps, the execution of the FATCAT step failed. The reason for the failure was that some of the PDB (protein) ids used in the input list had been superseded by newer structures in the PDB. Therefore, an additional component was added to check availability and replace any obsolete protein with its superseded version. This issue will not be unusual in reproducibility. Many experiments rely upon third party data sources that change constantly. Consequently, it is to be expected that these sources may not always be available and that the results that they return for the same given query may not always be the same. In our case, the changes in the PDB were addressed by adding a step that updated the older IDs with the new ones. **This suggests that some published results that depend on third party data sources may not always be reproducible exactly, so it would be good practice to publish all intermediate data from the experiment so that the method followed can be examined when re-execution is not possible. An alternative is that data archives provide access to their contents for each version.**

**Docking.** The raw interaction network resulting from the first subsection of the method (comparison of ligand binding sites) was assumed to be the input for docking. It turns out that although the input for docking is data produced by SMAP, it is not the raw interaction network that it outputs. Instead, it is data that SMAP places in an “alignment” folder - only expert users would be aware of this.

The original article refers to adding cofactors to relevant proteins prior to docking, which could be interpreted to be a step prior to docking. As it turns out, there is no explicit step for handling the cofactors since this is handled by manually editing the appropriate PDB file. Again, only expert users would be aware of this.

Examination of the author’s scripts revealed some additional steps: calculating the clip files, which are used for obtaining the ideal ligands before docking. Clip files are mentioned in the article as containing the aligned drug molecules, so it would seem to a non-expert that the aligned molecules would be the output of the initial alignment steps of the overall method.

A major issue with this portion of the workflow is that the docking software used for the original article was no longer used in the laboratory. It is proprietary software, and its license had expired, so alternative software (AutodockVina) with similar functionality has been adopted since the original article was published. Some of the ligands were not recognized by this software, so a transformation step had to be added to the workflow to make Autodock Vina work correctly.

There are reasons why authors use proprietary software, for example, ease of use, support, robustness, visualization and data types supported. However, the authors could replicate the method before publication using open source tools, which would facilitate reproducibility by others. **The use of open source software instead of proprietary software facilitates the reproduction of the software steps originally used by the authors, and should be the preferred mode of publication of methods and workflows.**

### Reproducibility Maps

**Figure 3:**
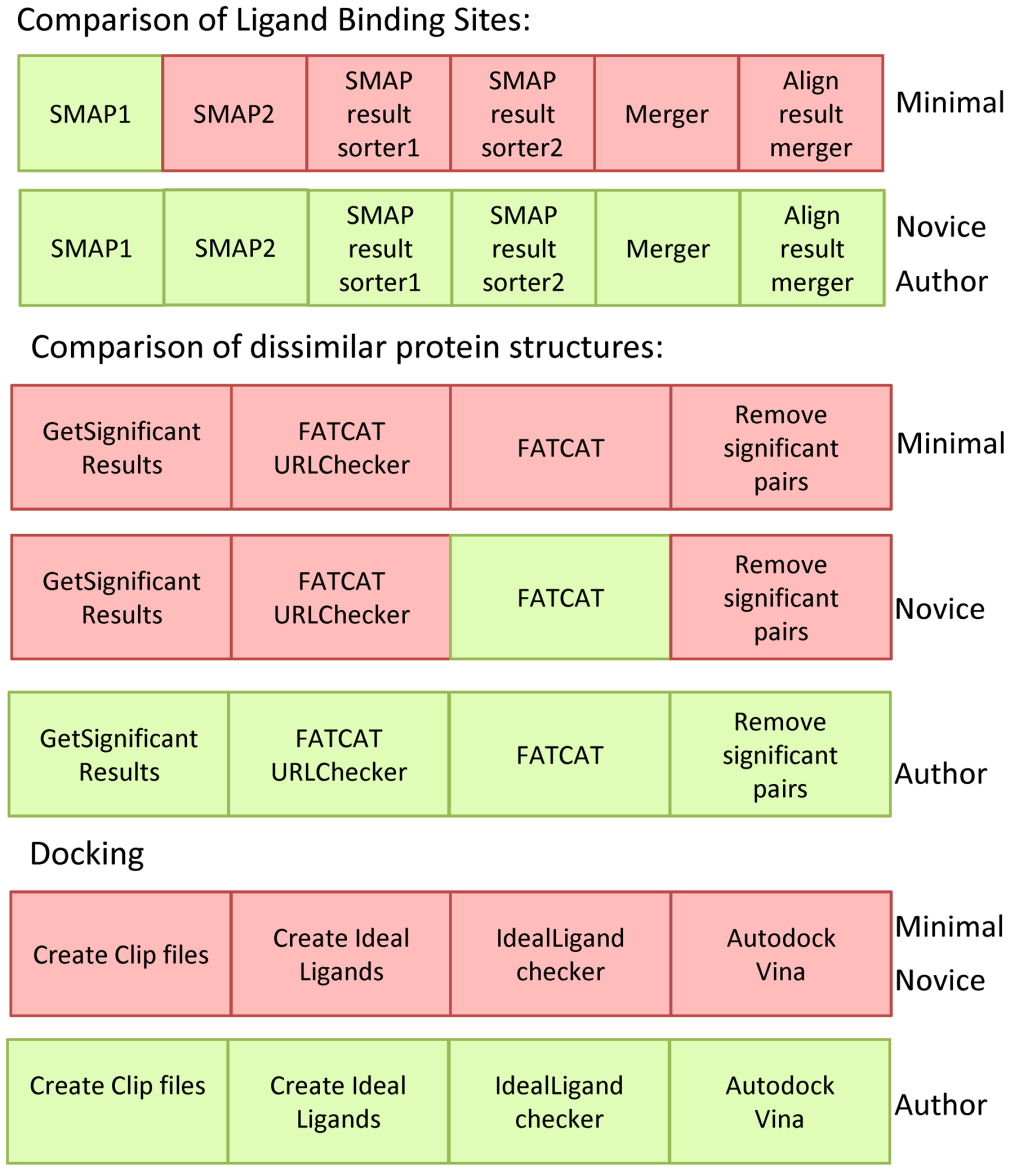
Reproducibility maps of the three major subsections of the workflow. A step is shown in red if it was not reproducible by that category of user, and green if it were.

We present reproducibility maps created as a summary of the reproducibility scores for all the major steps in the workflow. Figure 3 shows the reproducibility maps for each of the subsections, summarizing the reproducibility scores assigned to each step. For each section of the method, we show a progression of steps from left to right, noting on the right hand side the category of reproducer represented (MINIMAL, NOVICE, and AUTHOR). A step is shown in red if it was not reproducible by that category of user, and green if it were.

Our observation was that a researcher with minimal knowledge of the domain would only be able to reproduce one of the fourteen steps in the workflow. A novice researcher would be able to reproduce seven of the fourteen steps: the six steps to compare ligand binding sites, only one of the four steps to compare the protein structures, and none of the steps for docking. For docking, our conclusion was that only expert researchers with advanced knowledge of the domain would be able to reproduce the steps. The original software was no longer available, and advanced expertise was required to identify equivalent software to replace it, and to write the software necessary to make it work as needed. Expert researchers would be able to reproduce the method, as the original article combined with the data and software published in the site would be sufficient to infer any missing information. A detailed rationale for the scores can be found in the *reproducibility scores* subsection of Supplement S1.

Regarding the results, we checked that the output of the workflow included all the drugs exposed in the original work (plus new findings). The ranking of drugs in the results of the workflow is almost the same as the original, although the number of connections found for each drug is significantly higher in the results of the workflow. A possible reason is changes in the version of the software tools and updates to the external databases where the structures are stored. A detailed comparison can be seen in the *original results versus results from the workflow* subsection of Supplement S1.

### Productivity and Effort

We kept detailed records in a wiki of the effort involved in reproducing the method throughout the project. These records are publicly available from [36].

We estimated the overall time to reproduce the method as 280 hours for a novice with minimal expertise in bioinformatics. The effort included analyzing the paper and the original author’s web site and additional materials (data, scripts, configuration files) to understand the details of the method, locating and preparing the codes, finding appropriate parameter settings, implementing the workflows, asking questions to the authors when necessary, and validating the workflows. It should be noted that the authors of the original experiment were available to answer questions (notably Kinnings, the first author). These questions were related to missing configuration parameters, documentation for the proper invocation of the tools, and validation of the outcome of the intermediate steps. Table 1 estimates the time required to reproduce the method and is broken down by major tasks according to our records.

**Table 1:** Time to reproduce the method.

Tasks	Time (hours)
Familiarization with workflow and running software	160
SMAP steps	32
SMAP result sorter steps	8
Merger steps	4
Get significant results	4
FATCAT URL checker	8
FATCAT step	4
Remove significant pairs	4
Create clip files	8
Create ideal ligands	8
Ideal ligand checker	8
Autodock Vina	16
Data visualization steps	16
TOTAL	280 hours

### Publishing the Reproduced Workflow

Now that we had invested significant effort in reproducing the workflow, our goal was to maximize its reusability.

First, the executed workflow was published using the Open Provenance Model [32]. This model is used by many workflow systems, so it increases the workflow reusability because it can be imported into other systems depending on the preference of the particular research group. We also publish the workflow provenance using the PROV ontology [45], a recent standard for provenance from the W3C [46]. This makes the published workflow independent of the workflow system used to create it.

Second, we published an abstract workflow that complements it. The abstract workflow describes the steps in a manner that is independent of the software used to implement them. For this we used an extension of the Open Provenance Model called OPMW [47] that includes new terms to describe abstract steps.

Third, we published the workflow and all of its constituents (including input and output data, software and scripts for the steps) as Linked Data [48], which means that each constituent of the workflow can be accessed by its URI through HTTP, and its properties are described using W3C RDF standards [49]. This means that the published workflow is accessible over the Web, in a way that does not require figuring out how to access institutional catalogs or file systems.

With this maximally open form of publication of the workflow, the effort that we invested in reproducing the workflow does not have to be incurred by others. Each step and its inputs and outputs are explicitly and separately represented as well as linked to the workflow. The software for each step is available as well, as are the intermediate and final results.

The effort involved in creating a workflow is negligible compared with the time to implement the computational method. Implementing the computational method typically takes months, and involves activities such as finding software packages that implement some of the steps, figuring out how to set up the software (e.g., setting up parameters) to suit the data, and writing new code to reformat the data to fit those packages. Once this is all done, creating the workflow can be done in a few hours, and can be as simple as wrapping each step so it can be invoked as a software component and expressing the dataflow among the components. Learning to create simple workflows requires only a few hours, more advanced capabilities clearly require additional time investment (e.g., running workflows in a cluster, depositing results in a catalog, or expressing a complex control flow). Similarly, publishing workflows takes no effort at all since the workflow system takes care of the publication.

Technical details on how the workflow is published can be found in [50]. The OWL ontologies for OPM and PROV that express all the underlying RDF properties can be browsed from [51]. All the materials related to the workflow and its execution results have been published online [36]. Additionally, input and output datasets have been associated to DOIs and uploaded to a persistent data sharing repository [52].

## Discussion

Reproducibility is considered a cornerstone of the scientific method and yet rarely is scientific research reproducible without significant effort, if at all [5]-[7]. Authors submitting papers know this; as do those reading the papers and trying to reproduce the experiment. For computational work like that described here, where data, methods, and control parameters are all explicitly defined there is less of an excuse for not making the work reproducible. Note that making the software available or accessible through a webserver, while commendable, is not the same as making the work reproducible. Workflows, which define the scientific process as well as all the components, provide the tools for improved reproducibility. While workflows are commonly used for highly repetitive tasks, they are less used for earlier stage research. Whether this is a result of shortcomings in the tools or insufficient emphasis on the need to make work reproducible requires further consideration. This then raises the further issue of whether the emphasis itself is justified. Do we really care if work is *exactly* reproducible? This generally only becomes important if some variation of the original work cannot be reproduced at all, then the original work is fully scrutinized. This speaks to a need for better quantification of what is really needed to improve productivity in science. When, as is the case here, the experiment is conducted completely *in silico*, the opportunity to accurately capture what has transpired becomes a relatively straightforward task (i.e., there is a relatively favorable cost:benefit ratio) and raises the question as to whether the community of computational biologists should do better. What does doing better imply?

We believe it is rare that work is purposely made irreproducible; rather the system of peer review speaks to reproducibility but is cursory in demanding it. The scientific reward is in publishing another paper, not making your current paper more reproducible. Tools help, but changes in policy are also needed. It will be a brave publisher indeed that demands that workflows be deposited with the paper. Publishing after all is a business and if one publisher demands workflows, authors are more likely to publish elsewhere than go to the trouble. Journals are beginning to provide guidelines for reproducibility and minimum requirements for method descriptions [53]–[54]. There is already a concept of “data publication,” where datasets are described and receive a unique identifier and a publication. Similarly, there should be a concept of “workflow publication.” There is no explicit credit for publishing software packages, and many people do it. The credit comes indirectly from acknowledgement by the community that the software is useful. Perhaps publishing end-to-end methods as workflows would bring similar reputation. For this to work, authors must be recognized and credited by other researchers reusing their workflow. We posit that the authors of the original method need not be the ones publishing the workflow. Third parties interested in reproducing the method could publish the workflow once reproduced, and get credit not for the method but for the workflow as a reusable software instrument. In one sense this is no different than taking other scientists data and developing a database that extends the use of these data to a wider community. It is a value-added service worthy of attention through publication.

Federal mandates similar to those emerging around shared data could also be put in place for reproducibility too. In the end, funding for science ultimately comes from taxes from the public, and we need to be responsible in making science as efficient and productive as possible. Many government agencies already require data to be published and shared with other researchers. Workflows should follow the same path. The recent emphasis on open availability of research products resulting from public funds [55]–[56] will eventually include the publication of software and the methods (workflows). This will likely be sometime coming as the easier issue of meaningful data provision is not fully understood and solved yet. Notwithstanding, if this remains a difficult issue on a global scale we can make progress in our own laboratories.

A new researcher coming to almost any laboratory and picking up tools used by previous laboratory members can likely testify to what is described in this paper. If we are to accelerate scientific discovery we must surely do better both within a laboratory and beyond. This is particularly important in an era of interdisciplinary science where we often wish to apply methods that we are not experts in. Some would argue that irreproducibility in the laboratory is part of the learning process; we would argue yes, but with so much to learn that is more relevant to discovery we should do better now that we have tools to assist us.

Or should we? Reproducibility aside, is there indeed a favorable cost:benefit ratio in using workflows with respect to productivity? There is a dearth of literature that addresses this question. Rather the value of the workflow is assumed and different workflow systems on different computer architectures are analyzed for their relative performance. At best the question can be addressed by work habits. We must be careful as such work habits could be mandated, in a large company say, rather than by choice, which would be the case in an independent research laboratory. Creating workflows results in overhead for exploratory research, where many paths are discarded. However, once created a workflow can be reused many times. This makes them ideal for repetitive procedures such as might be found in aspects of the pharmaceutical industry. Pharmaceutical companies use workflows for computational experiments [57]. This means there must be a business case for workflows in terms of saving time and effort and/or facilitating quality control. Taking an independent computational biology laboratory, as is the case for this study, it is fair to say that workflows are making inroads into daily work habits. These inroads are still localized to specific subareas of study – Galaxy [58] for high-throughput genomic sequence analysis; KNIME [59] for high-throughput drug screening, and so on, but with that nucleation and with new applications being added by an open source-minded community, adoption is increasing. Adoption would assume a favorable cost:benefit ratio in that use of a workflow system provides increased productivity over not using such a system. This is a cost measured in time rather than money since most academic laboratories in computational biology would use free open source workflow systems. Finally, when articles cannot be easily reproduced the authors are often contacted to clarify or describe additional details. This requires effort that might as well have been invested in writing the article more precisely in the first place.

Workflows can also be seen as an important tool to make the research in a lab more rigorous. Analyses must be captured so they can be inspected by others and errors detected as easily as possible. For example, writing code to transform data makes the transformation inspectable, while using a spreadsheet to do the task makes it much harder to verify that it was done correctly. Ensuring consistency and reproducibility requires more effort without workflows. In our own laboratory we find that the workflow can act as a reference such that new users can more quickly familiarize themselves with the various applications than would be the case without the benefit of the workflow organization, but then choose to go on and run applications outside of the workflow system. As the workflow systems themselves continue to be easier to use and more intuitive we anticipate that more work will be done within the workflow system itself, presumably improving productivity.

For the practitioner, what are the pluses and minuses of workflow use today? An obvious minus is the time required to establish the workflow itself. In some sense this is analogous to documenting a procedure to run a set of software programs. But in most cases once codes are prepared for publication little additional effort is required to include them in a workflow. The advantage of a workflow is that capturing the steps themselves defines the procedure and it can be re-run, in principle, without any further effort. We say “in principle” since as this work has shown workflows decay – the tools available change, the licenses to those tools change, remote data accessibility changes etc. Virtual machines offer the promise of capturing the complete executable environment for future use, however they introduce other issues [26]. For example, virtual machines often act as black boxes that allow repeating the experiment verbatim, but do not allow for any changes to the computational execution pipeline, limiting its reproducibility. Furthermore, virtual machines cannot store external dynamic databases accessed at runtime (like the PDB in our work) due to their size. These databases are commonly used for experiments in computational biology.

All taken together, it may be that we are at this tipping point of broad workflow adoption and it will be interesting to review workflow use by the computational biology community two or more years from now.

## Conclusions

We conclude by summarizing the main observations resulting from our work, leading to desiderata for reproducibility shown in Table 2, and a set of guidelines for authors shown in Table 3. We have restrained from making too many absolute conclusions from a single instance of applying a workflow to a scientific method. It would be interesting to carry out similar studies in other domains and compare findings.

**Table 2:** Observations and desiderata for reproducibility.

Observation
·We found that important computational steps were either missing or ambiguous. **The paper should make clear all computational steps needed by a novice user.**
·Software is often used with carefully selected parameter settings and configurations. **It would be good practice for authors to publish not just a description of the software and data used, but also to publish any parameter settings and configuration files used.**
·The possibility of re-running the method periodically with new versions of software tools leading to new findings might help entice researchers to keep their methods readily reproducible.
·Published results that depend on third party data sources may not always be accessible and may make the experiments run by the original authors irreproducible. **Where practical, authors should publish all intermediate data from the experiment so that the method they followed can be examined when direct re-execution is not possible.**
·To implement some steps of their methods, authors often use proprietary software or software that is not widely available. **The use of open source software facilitates the reproduction of the software steps originally used by the authors, and should be the preferred mode of publication for authors of methods and workflows.**
·Although many methods are implemented by using public domain software tools, they often contain additional steps that were implemented by the authors. **To facilitate reproducibility, authors should publish any software written by them and that became part of the method.**

**Table 3:** Reproducibility Guidelines for Authors.

Guideline
1. **Input data:** Provide the original datasets used in the experiment reported in the paper
2. **Dataflow Diagram:** Provide a diagram that represents a dataflow of the computational steps. The nodes in the graph should be computational steps, which include invocations of software tools, scripts and other software that were written, and any additional data manipulations that were carried out manually. The links in the graph specify the dataflow, which indicates what the input data for each step are and links to other steps that may have generated the data.
3. **Software:** Prefer open software tools that are appropriately documented. Specify the software tools used mentioning versions and download dates. For any scripts or other software that were written, provide the code itself or at least “pseudo-code” (i.e., an informal version of the code that is language-independent)
4. **Configurations:** Provide the values of any parameters and configuration files used
5. **Intermediate data:** Provide key intermediate data that resulted from important steps and that would help others determine whether they reproduced the method correctly

## Supporting Information

Supplement S1 **A detailed account of the Reproducibility of the TB Drugome Method.**
(DOCX)
