# Supplementary material for: Quantifying Reproducibility in Computational Biology: The Case of the Tuberculosis Drugome
Source: PLoS One. 2013 Nov 27;8(11):e80278. doi: 10.1371/journal.pone.0080278 (PMC3842296; doi:10.1371/journal.pone.0080278)
Supplement: Supplement S1 — A detailed account of the Reproducibility of the TB Drugome Method. (DOCX) [file pone.0080278.s001.docx]

# Supplement S1. A Detailed Account of the Reproducibility of the TB Drugome Method

We describe the evolution of the reproduction of the method as a series of conceptual diagrams resulting from the interaction between the computer scientist and the authors while gathering more information. Finally, we show the method represented as a workflow of computational steps. We also show reproducibility scores for each of the steps of the resulting workflow.

### Comparison of Ligand Binding sites using SMAP

Relevant paragraph of methods section from [Kinnings et al. 2010]:

*“Xie et al. recently developed the ligand binding site comparison software SMAP [22], which is based on a sequence order independent profile-profile alignment (SOIPPA) algorithm [24]. Firstly, the protein structure is characterized by a geometric potential; a shape descriptor that is analogous to surface electrostatic potential, but which uses a reduced C-alpha only structural representation of the protein. It has been shown that both the location and the boundary of the ligand binding site can be accurately predicted using the geometric potential [23]. The reduced representation of the protein structure makes the algorithm tolerant to protein flexibility and experimental uncertainty; thus SMAP can be applied to low-resolution structures and homology models. Secondly, two protein structures are aligned, independent of sequence order, using a fast, maximum weighted sub-graph (MWSG) algorithm [80], [81]. The MWSG finds the most similar local structures in the spirit of local sequence alignment. Finally, the aligned surface patches are ranked by a scoring function that combines evolutionary, geometric and physical information. The statistical significance of the binding site similarity is then rapidly computed using a unified statistical model derived from an extreme value distribution [22].*

*The SMAP software was used to compare the binding sites of the 749 M.tb protein structures plus 1,446 homology models (a total of 2,195 protein structures) with the 962 binding sites of 274 approved drugs, in an all-against-all manner. While the binding sites of the approved drugs were already defined by the bound ligand, the entire protein surface of each of the 2,195 M.tb protein structures was scanned in order to identify alternative binding sites. For each pairwise comparison, a P-value representing the significance of the binding site similarity was calculated.”*

#### Uncovering the Workflow

**First attempt:** The first sentence of the second paragraph was interpreted as using the SMAP software as a single step to compare the three items: the binding sites, the homology models, and the drugs. Therefore the first diagram of this workflow fragment can be seen in Figure S1:

Figure S1: First attempt to reproduce the SMAP analysis

**Second attempt:** The SMAP software takes only two source arguments, so it became clear that there were two separate executions of the SMAP software: one to compare the binding sites of experimental structures with the drug-receptor binding sites, and one to compare the homology model binding sites with the drug-receptor binding sites. Note that the two SMAP steps can be run independently, but both invoke the same SMAP software. Another important clarification was that the tables were not the direct input to the SMAP software. Therefore, it was assumed that some processing of the tables occurred to prepare the data for SMAP. A second diagram (shown in Figure S2) was created:

Figure S2: Second attempt to reproduce the SMAP analysis

**Third attempt:** Examining the SMAP software, it became clear that the input to the SMAP components were not the tables in the paper but lists of solved structures and homology models obtained from queries to the PDB and other biomedical websites. As it often happens, the tables were derived from the actual data that were analyzed and were not the direct input to the workflow. Figure S3 summarizes the third attempt to reproduce the workflow fragment.

Figure S3: Third attempt to reproduce the SMAP analysis

**Fourth attempt:** After becoming familiar with SMAP, the output of the tool became clear. By inspecting the script that invoked the SMAP tool, it became apparent that there was a sorting step. An examination of the code revealed that the results of SMAP were not just sorted, but that all the results below a given p-value were filtered out. The following diagram (shown in Figure S4) was created:

Figure S4: Fourth attempt to reproduce the SMAP analysis

#### Computational Workflow

The final workflow that represents all the computational steps has been depicted in Figure S5:

Figure S5: SMAP workflow

The left strand of the workflow processes and filters the solved structure comparisons against the drug binding sites, while the right strand of the workflow computes and filters the homology models. A final step, Merger, is used to merge both outputs as a whole new dataset.

There are several things to note in the workflow. First, the p-value parameter is not needed for the SMAP step, only for the sorting step, but it was preserved as input to the SMAP step because both the SMAP and sorting steps were in the same original step, and it was decided to preserve its structure (the p-value was an input to that step). Second, the drug binding sites are input to the sorting steps. This was exposed when the software were examined. The drug binding sites are only used to extract the names of the drugs. Third, the solved structures and homology models are input to the respective sorting steps, being used for extracting the names of the proteins and homology models.

There are also other issues that arise that will be of no surprise to those who have previously attempted to reproduce the work of others based on a research article alone. One issue had to do with the publication of configuration parameters for tools used in a method. The SMAP software has a configuration file as input. This includes the p-value used, (that could be different than the one used for the threshold of the sorting step), *MATCH_SECONDARY_STRUCTURE, LIGAND_CONTACT_DISTANCE_CUTOFF, TEMPLATE_LIGAND_SITE_ONLY, TEMPLATE_LIGAND_ID, ASSOCIATE_GRAPH_NODE_FILTER, TIMES_RANDOM_SHUFFLE*, and so on. Without the authors configuration files used, default values of the parameters were used not knowing if the workflow would produce questionable results. That is, it is not clear whether without the same parameter settings the original method would be reproduced and similar results would be obtained. For these reasons, the original configuration files were obtained from the authors. **This suggests that it would be good practice for authors to publish not just a description of the software used and the data used in the original experiment, but also the configuration files used.**

Another issue concerned the constant evolution of the software tools that are used for the method steps. In our case, the SMAP software had evolved since the publication of the original paper. As with many software tools used in biology, SMAP is an active research effort and its functionality continues to improve. When the workflow was reproduced there was a new version of SMAP that had the same basic functionality but different input requirements. As a result, the intermediate and final data generated by the workflow were not exactly the same as the original results. This is not an unusual situation, as software tools are continuously improved over time. Under normal research circumstances, it is not critical that the workflow reproduce the experiment exactly. What is important is that the original results stand the test of time, and any significant findings are still significant no matter what tools are used. An interesting result would be if the workflow was run again with a newer more powerful tool and there were additional findings over and above the original publication. The same can be said for new and more comprehensive sources of input data. **The possibility of easily re-running the method periodically with new versions of software tools and/or data that might lead to additional findings may entice researchers to keep their methods more readily reproducible.**

#### Reproducibility Scores

Table S1 summarizes our reproducibility scores of this workflow fragment. The last column of the table is a brief justification of the scores. Some of the justifications simply show a quote from the original article where the step is mentioned, highlighting text that refers to inputs or outputs of the step.

We start with the SMAP1 step. First, we assign a score based on whether the existence of that step can be determined. In this case, it is mentioned in the original article, so the score is a “1” in the category MINIMAL for the existence of the step. Next, we consider whether the software for that step (which we call a software component) can be identified. The article mentions the SMAP software, so the score is a “1”. The inputs are mentioned in the article, so the scores are “1” for MINIMAL. The configuration parameters are not specified in the article, so the score is “0” for MINIMAL. One has to investigate the SMAP software, look at its default configuration, and test whether that works, which requires basic knowledge of the domain. So we assigned a score of “1” for NOVICE. The outputs of the step are mentioned in the article, so the score is “1” for basic.

The SMAP2 step, which deals with the homology models, is not mentioned as a separate step in the original article. So it has a score of “0” for MINIMAL and a score of “1” for NOVICE, since looking at the SMAP software reveals how to use it with different inputs. The rest of the reproducibility scores are analogous to the SMAP1 step.

The SMAPResultSorter1 step is not explicitly mentioned in the article, so it receives a score of “0” for MINIMAL. Anyone with basic knowledge of the domain would infer that sorting was necessary, so it has a score of “1” for NOVICE.

The Mergers step are not mentioned in the article, to the score assigned is “0” for MINIMAL. The need to merge results is obvious once the two SMAP steps are done, so the score is “1” for NOVICE.

Table S1: Reproducibility scores for the SMAP workflow

### Comparison of dissimilar protein structures using FATCAT

Relevant paragraph of methods section from [Kinnings et al. 2010]:

*“FATCAT (Flexible structure AlignmenT by Chaining Aligned fragment pairs allowing Twists)* [*[82]*](http://www.ploscompbiol.org/article/info%3Adoi%2F10.1371%2Fjournal.pcbi.1000976#pcbi.1000976-Ye1) *is a program for the flexible comparison of protein structures. It optimizes the alignment between two structures, whilst minimizing the number of rigid body movements (twists) around pivot points introduced in the reference structure. In addition to the optimal structural alignment, FATCAT reports the statistical significance of the structural similarity, measured as a P-value. In order to identify pairs of similar binding sites that were from proteins with dissimilar global structures (i.e., cross-fold connections), the first chain of each PDB file was aligned using FATCAT, and those pairs with a significant P-value of less than 0.05 were discarded.”*

#### Uncovering the Workflow

**First attempt:** The first interpretation of the paragraph clearly led to two different components: the FATCAT tool plus a filtering step for removing the p-values of less than 0.05. What were not that clear were the inputs to each component: it was interpreted as if FATCAT produced an output which was used together with the SMAP output for the filtering step. Figure S6 shows the first attempt to reproduce FATCAT workflow fragment.

Figure S6: First attempt to reproduce the FATCAT analysis

**Second attempt:** After having access to the scripts used by the authors, it became clear that FATCAT uses as input a list of pairs of proteins regardless of whether they were experimentally determined or homology models. So the first part of the workflow (first two ovals and first box in the previous figure), would be replaced by Figure S7:

Figure S7: Second attempt to reproduce the FATCAT analysis

**Third attempt:** The list used as input to FATCAT is computed through an additional step, not mentioned in the paper. After having access to the scripts of the authors it was clear that it produces another additional list, called “significant results”. None of these inputs and processing steps are mentioned in the paper.

#### Computational Workflow

The WINGS workflow represents the steps as the workflow was finally defined (Figure S8).

Figure S8: FATCAT workflow

Note that checkedList is an input to FATCAT. However, the article refers to “the first chain of each PDB file was aligned using FATCAT”. The article does not state explicitly that the checkedList is in fact from the PDB.

The steps RemoveSigPairsFATCAT and GetSignificantResults are not described in the article. An expert in the domain would infer the need for these steps from the published article, looking at the passage: “identify pairs of similar binding sites.”

In addition, the article mentions a threshold of 0.05 that does not appear anywhere in the workflow: “and those pairs with a significant P-value of less than 0.05 were discarded.” This ended up being a default value in FATCAT, so it does not need to be explicitly set up. An expert would understand that this is the default value by reading the FATCAT documentation.

An important issue for reproducibility came up in this portion of the workflow. Although the method was reproduced with all of the necessary steps, the execution of the FATCAT step failed. The reason for the failure was that some of the protein ids used in the input list had been superseded by other structures in the PDB. Therefore, an additional component was added to check availability and replace any possible protein with its superseded version. This component was named FATCATURLChecker.

This issue will not be unusual in reproducibility. Many experiments rely upon third party data sources that change constantly. Consequently, it is to be expected that these sources may not always be available and that the results that they return for the same given query may not always be the same. In our case, the changes in the PDB were addressed by adding a step that updated the older IDs into the new ones. **This suggests that some published results that depend on third party data sources may not always be reproducible exactly as the original authors ran the experiment, so it would be good practice to publish all intermediate data from the experiment so that the method followed can be examined when re-execution is not possible.**

#### Reproducibility Scores

Table S2 shows the reproducibility scores for this workflow fragment. The first two steps were not mentioned in the original article. Novices would not be able to determine the need for these steps. Only experts would, so the score is “1” for AUTHOR.

The FATCAT step is mentioned in the original article. However, its inputs are mentioned in an ambiguous manner in the text so some degree of expertise is required. For this reason, we assigned a score of “0” for MINIMAL and “1” for NOVICE.

The RemoveSigPairsFATCAT step is not mentioned in the original article. It requires some basic expertise to determine how to combine the results of the two previous comparisons from SMAP and FATCAT. Therefore we assigned a score of “0” for MINIMAL and “1” for NOVICE.

Table S2: Reproducibility scores for the FATCAT workflow

An important issue that came up with this portion of the workflow is that there were two steps (GetSignificantResults and GetSignificantPairsFATCAT) that were implemented by the authors as scripts that were not initially available. The workflow would have been harder to reproduce without those scripts. Once we had obtained the scripts from the authors, the workflow was easily completed. Note that these scripts are not available in the paper or in the accompanying website. **Authors should be encouraged to publish any software that were written by them and that became part of the method, because public domain software tools do not provide all of the software required to reproduce the method.**

### Docking using eHits/AutodockVina

Relevant paragraph of methods section from [Kinnings et al. 2010]:

*“For those pairs of interest, molecular docking was used to predict the binding pose and affinity of the drug molecule to the M.tb protein. eHiTS Lightning* [*[84]*](http://www.ploscompbiol.org/article/info%3Adoi%2F10.1371%2Fjournal.pcbi.1000976#pcbi.1000976-Zsoldos1) *was selected due to its fast speed, relatively high accuracy and ease of automation for large-scale docking studies. Since SMAP had aligned the drug binding site with the M.tb protein binding site, the aligned coordinates of the drug molecule were used to define the search space for docking that drug into the M.tb protein. The aligned drug molecule was used as the clip file with a default search space of 10Å3. As recommended by the manual, the eHiTS accuracy level was set to 6 (default = 3), in order to increase the accuracy of the predicted binding poses. Following all docking, the binding pose with the lowest estimated binding affinity was selected for further investigation. For those proteins with cofactors (e.g., InhA has an NAD cofactor), the cofactor was added as the last residue in the protein structure prior to docking.”*

#### Uncovering the Workflow

**First attempt:** The SMAP Output (i.e., the rawInteractionNetwork) was assumed to be the input for this step. However, the inputs used are data produced by SMAP, which places the data in an “alignment” folder. In addition, it seemed that a cofactor step was a component of the workflow: “For those proteins with cofactors, the cofactor was added as the last residue in the protein structure prior to docking.” Figure S9 summarizes the first attempt.

Figure S9: First attempt for reproducing the docking analysis

**Second attempt:** As it turns out, there is no explicit step for handling the cofactors. In addition, after email discussions with the authors and having access to the scripts, the workflow turned out to have some additional intermediate steps: calculating the clip files, which were used for obtaining the ideal ligands. Clip files are mentioned in the article (“The aligned drug molecule was used as the clip file with a default search space”…), but they seemed to be part of the SMAP output too.

**Third attempt:** A major issue with this portion of the workflow is that the eHits tool was no longer used in the laboratory. eHits is proprietary, and its license had expired. The new tool being used was AutodockVina, which has similar functionality. The IdealLigandCheckerImpl was added to obtain the docking results. Some of the ligands created by the CreateIdealLigandsImpl were not recognized in the AutodockVina step, so a conversion step had to be created for some of them. It could be considered a formatting step. If this step had not been included, this portion of the method would not have been reproduced. Figure S10 shows the final workflow developed in this step.

#### Computational Workflow

Figure S10: Docking workflow

There are other possible alternatives for this workflow. ClipFiles could be input to AutodockVina, so that the create and check ligand steps would not be needed. Another change is that GetSMAPAlignmentFolder could be eliminated if the data is already extracted from the folders where SMAP outputs the data.

#### Reproducibility Scores

The reproducibility of this subsection of the method was greatly affected by the change to a new software (Autodock Vina instead of eHits). Therefore, the reproducibility scores shown in Table S3 reflect that more advanced expertise would be required to adapt the method to use the new software. Most steps have a score of “0” for MINIMAL and most only reach “1” for the AUTHOR level. Although some steps are mentioned in the article (e.g., docking), advanced expertise is required to implement the software needed to adapt the method to use the software, so for the software component the score is “0” for MINIMAL and NOVICE and “1” for AUTHOR. For example, the docking step is mentioned in the article, and the software as well (eHits), but if the original software is not available then advanced expertise would be required to identify equivalent software (e.g., to know that AutodockVina is an appropriate substitute) and to use it correctly.

An additional step that the new software required was IdealLigandCheckerImpl. This is because Autodock Vina did not recognize some of the ligand identifiers. Recognizing the need for this step, and having the ability to create the configuration scripts requires advanced expertise.

Table S3: Reproducibility scores for the docking workflow

The reproducers were not able to reproduce this sub-workflow just from the paper, since it required figuring out how to implement it using the Autodock Vina software. Additionally, there were scripts developed by the authors that eventually were available to the reproducers. By looking at the paper and the documentation of the Autodock Vina tool, the reproducers were not able to figure out any of the steps in this workflow strand. Once the reproducers had access to the scripts originally used by the authors, they were able to set up most of the workflow. The reproducers did need further assistance from the authors in building the checker.

An important issue concerning reproducibility was exposed with this sub-workflow. The software tools used in the original experiments by the authors may not be available to the reproducers. One possible reason, as it was in our case, is that the authors used proprietary software to implement some of the method steps. There are reasons why authors use proprietary software, for example, ease of use, support, robustness, visualization and data types supported. However, the authors could replicate the method before publication using open source tools, which would facilitate reproducibility by others. **The use of open source software facilitates the reproduction of the software steps originally used by the authors, and should be the preferred mode of publication of methods and workflows.**

#### Original results versus results from the workflow

#### The summary of the results obtained in the original work can be seen in Table S4, while the results from the workflow are shown in Table S5. Both tables show a ranking of the highly connected drugs (ordered by their number of connections) and the M.tb proteins with solved structures found within those connections. As we can see in the tables, all the drugs belonging to the ranking in the original results have been found in the results of the workflow. The only difference between them is the number of connections (higher in the results of the workflow), and the fact that new highly connected drugs appear in the ranking. After discussing the results from the workflow with the authors of the original article, we can claim that we have successfully reproduced the method. The difference between the results is reasonable, as a different version of the software was used for some of the steps (SMAP) and the external databases (such as the PDB, where the structure of the proteins is stored) are dynamic. Figure S11 shows the drug-protein network obtained as a result from the workflow. The size of each drug node in the figure is proportional to its number of connections.

Table S4: Highly connected drugs, original results

Table S5: Highly connected drugs, results of the workflow. The entries found in the original results have been highlighted showing the commonalities between both

**Figure S11: Visualization of the drug-protein network obtained as a result of the workflow**

**FIGURES AND TABLES**

**
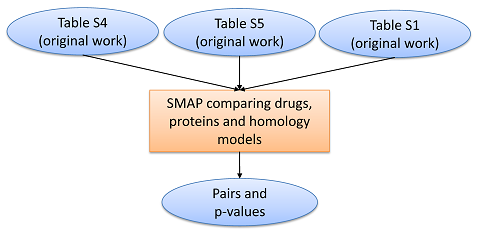
**

Figure S1: First attempt to reproduce the SMAP analysis


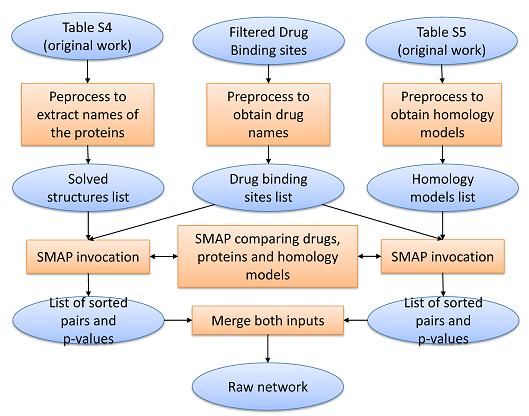


Figure S2: Second attempt to reproduce the SMAP analysis


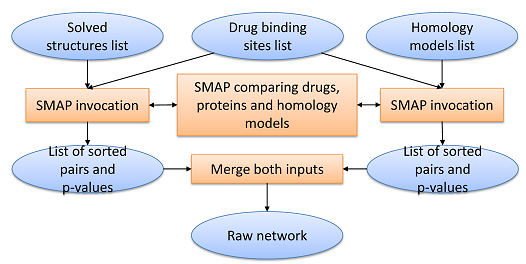


Figure S3: Third attempt to reproduce the SMAP analysis


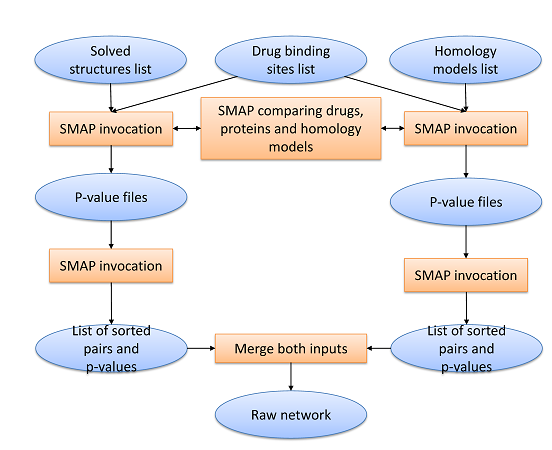


Figure S4: Fourth attempt to reproduce the SMAP analysis


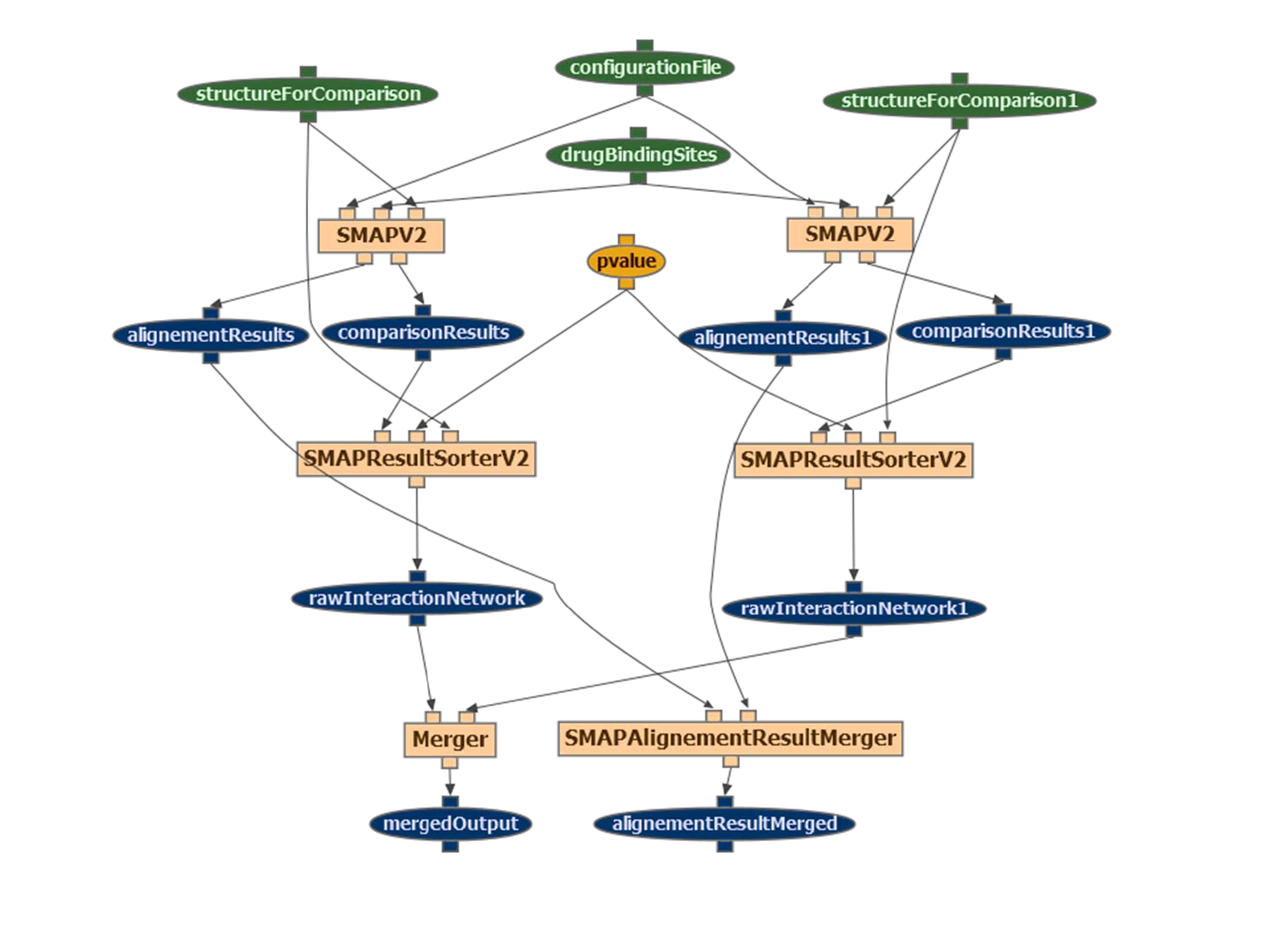


Figure S5: SMAP workflow


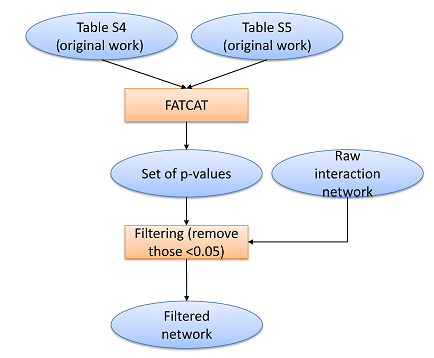


Figure S6: First attempt to reproduce the FATCAT analysis


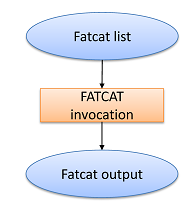


Figure S7: Second attempt to reproduce the FATCAT analysis


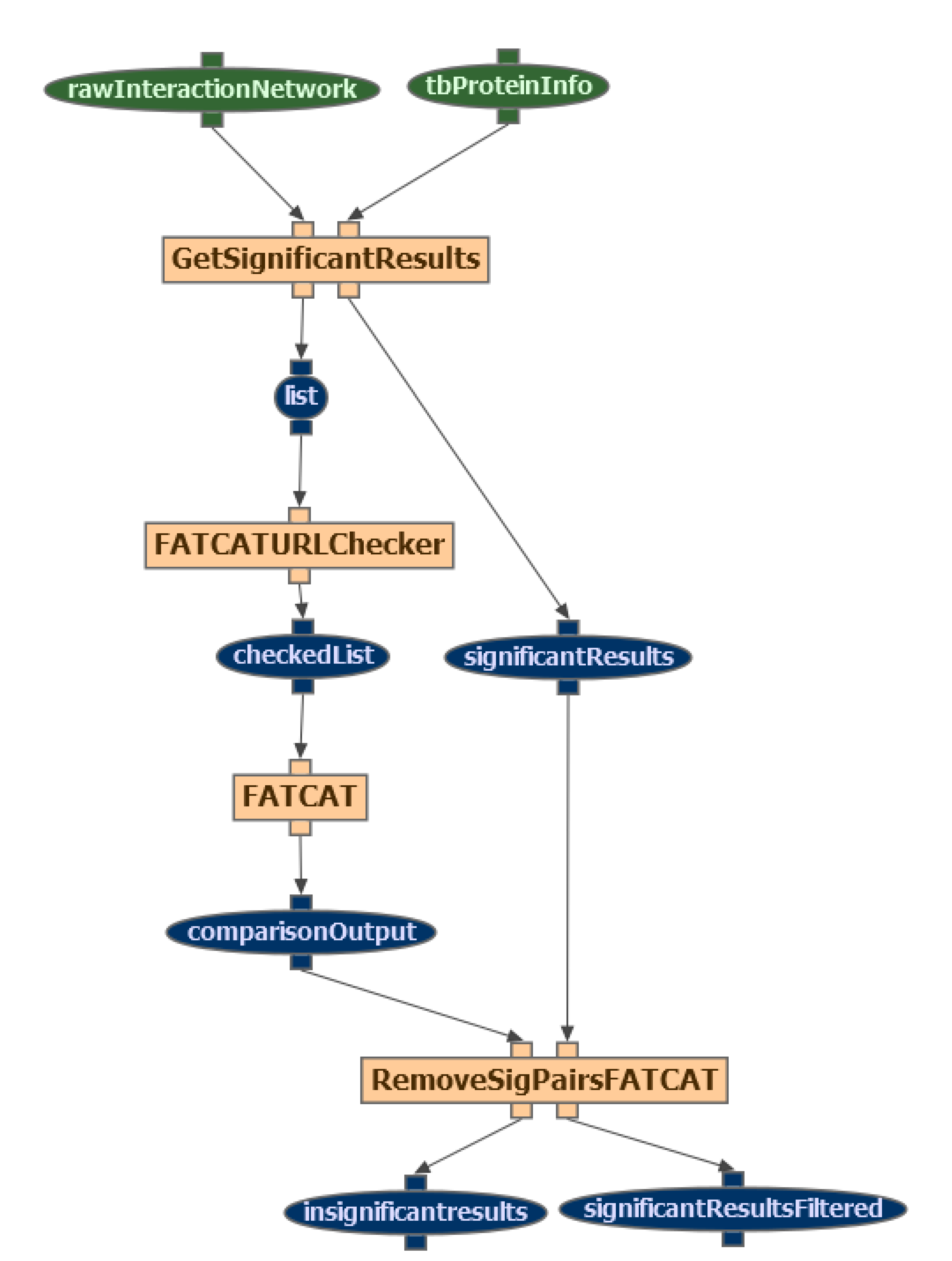


Figure S8: FATCAT workflow


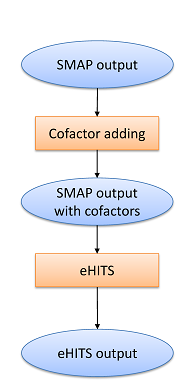


Figure S9: First attempt for reproducing the docking analysis


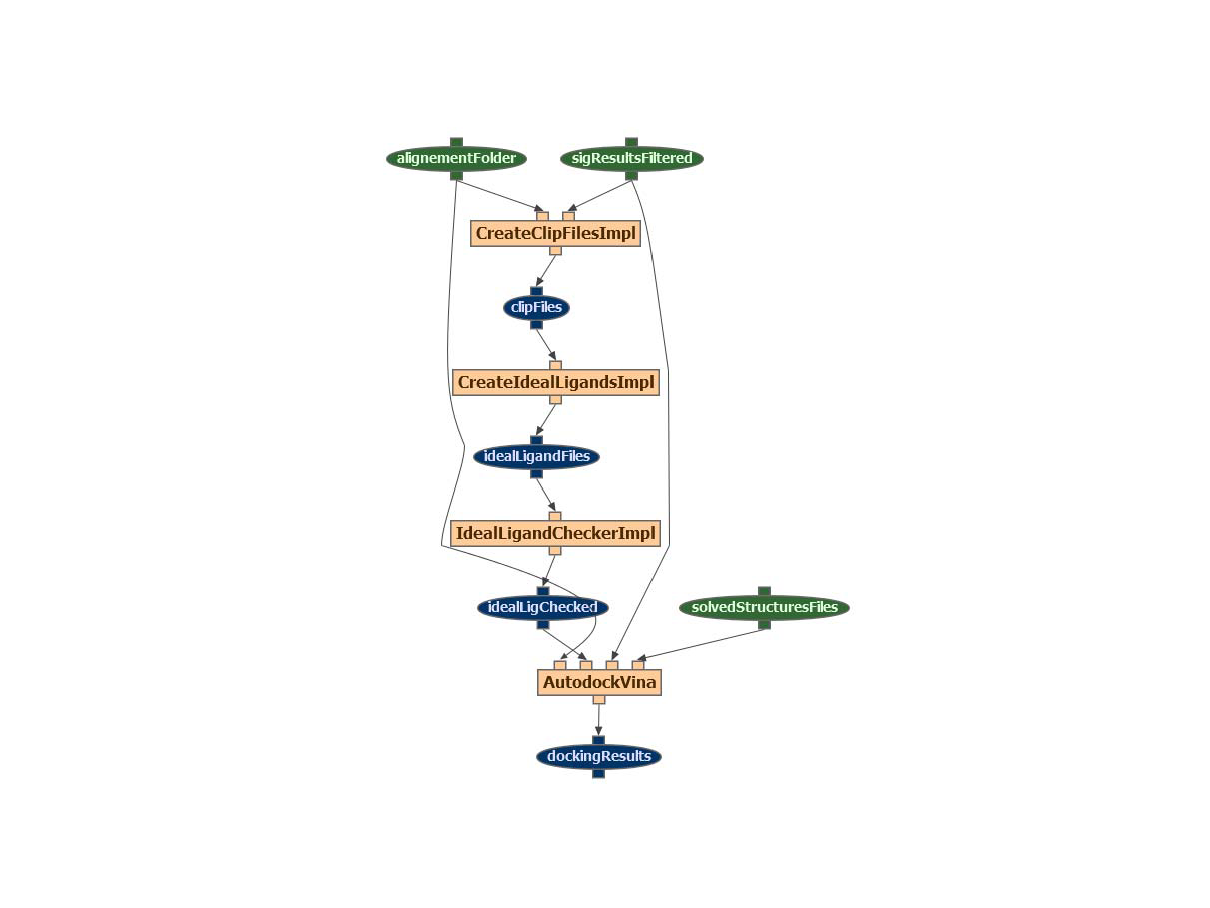


Figure S10: Docking workflow


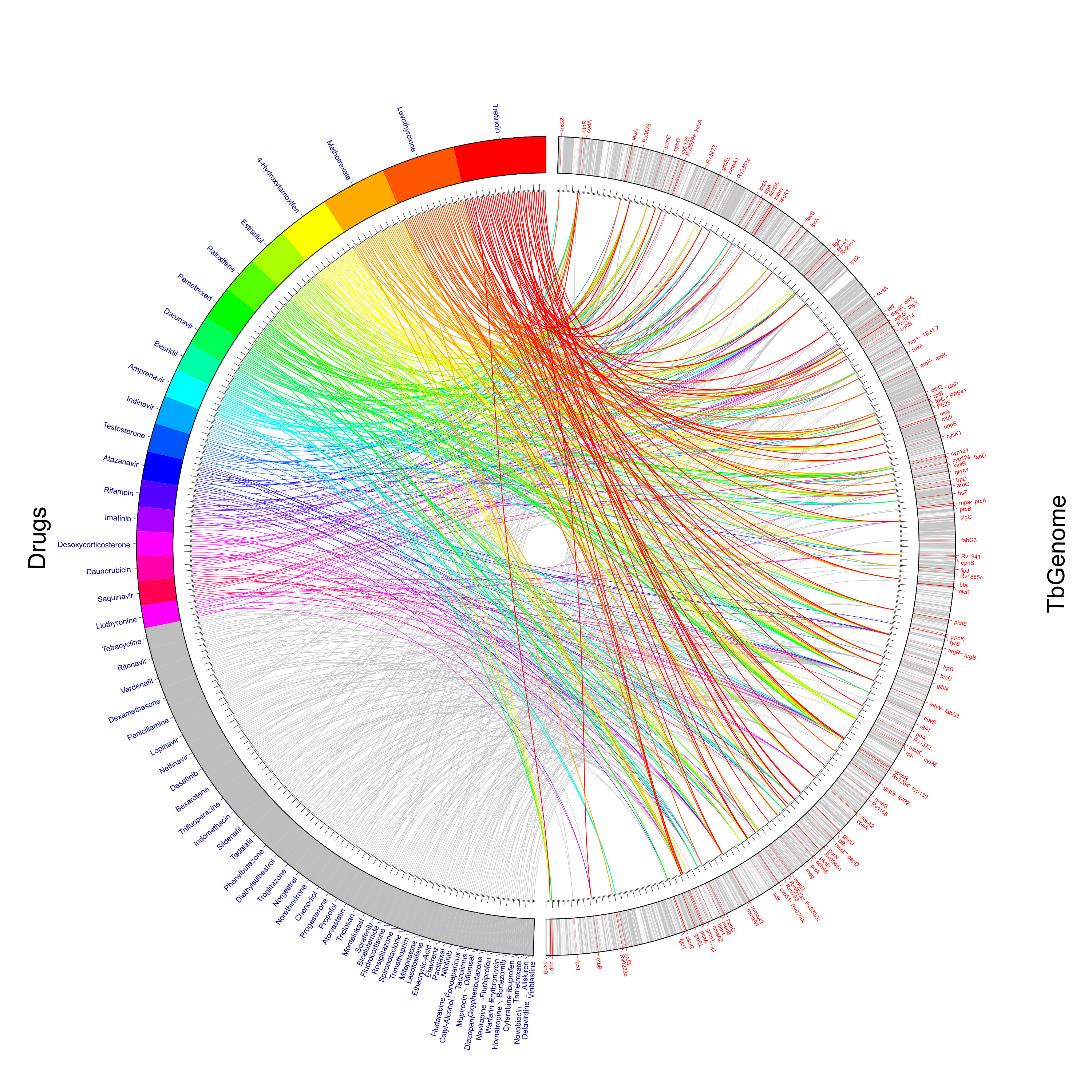


**Figure S11: Visualization of the drug-protein network obtained as a result of the workflow**

**Table S1: Reproducibility scores for the SMAP workflow**

| **Workflow Component** | **MINIMAL** | **NOVICE** | **AUTHOR** | **Justification** |
| --- | --- | --- | --- | --- |
| **SMAP1 step** | 1 | N/A | N/A | “The SMAP software was used to compare the binding sites of the **749 protein structures** plus the 1446 homology models with the **962 binding** sites of 274 approved drugs” |
| software component | 1 | N/A | N/A |  |
| input1: ligand binding sites | 1 | N/A | N/A |  |
| input2: drug binding site | 1 | N/A | N/A |  |
| input3: configuration parameters | 0 | 1 | N/A | The original article does not mention configuration parameters. However, the default values of the parameters in the SMAP software seemed to work. |
| output1: ComparisonResults | 1 | N/A | N/A | “For each pairwise comparison **a P-value representing the significance of the binding site similarity was calculated**” (the result of the component is a p-value) |
| output2: AlignementResults | 1 | N/A | N/A | “Secondly, two protein structures are aligned, independent of sequence order…” |
| **SMAP2 step** | 0 | 1 | N/A | “The SMAP software was used to compare […] **1446 homology model**s with the **962 binding sites** of 274 approved drugs”. However, we have given a 0 in MINIMAL because reading it in the paper it looks like it is a single step. |
| software component | 1 | N/A | N/A |  |
| input1: homology models | 1 | N/A | N/A |  |
| input2: drug binding site | 1 | N/A | N/A |  |
| input3: configurationParameters | 0 | 1 | N/A | The original article does not mention configuration parameters. However, the default values of the parameters in the SMAP software seemed to work. |
| output1: ComparisonResults1 | 1 | N/A | N/A | For each pairwise comparison, a P-value representing the **significance of the binding site similarity was calculated** |
| output2: AlignementResults | 1 | N/A | N/A | “Secondly, two protein structures are aligned, independent of sequence order…” |
| **SMAPResultSorter1 step** | 0 | 1 | N/A | In the paper there is no reference to a possible sorting of the SMAP results. However, further exploration of the author’s scripts showed that each p-value is filtered and sorted for each structure for comparison (as a list) instead of having each comparison in an individual file. |
| software component | 0 | 1 | N/A |  |
| input1: drugBindingSites | 0 | 1 | N/A |  |
| input2: ComparisonResults | 0 | 1 | N/A |  |
| input3: pvalue | 0 | 1 | N/A |  |
| input4: structureForComparison | 0 | 1 | N/A |  |
| output1: RawInteractionNetwork | 0 | 1 | N/A |  |
| **SMAPResultSorter2 step** | 0 | 1 | N/A | The rationale is the same as the SMAPResultSorter1 step. |
| component | 0 | 1 | N/A |  |
| input1: drugBindingSites | 0 | 1 | N/A |  |
| input2: ComparisonResults | 0 | 1 | N/A |  |
| input3: pvalue | 0 | 1 | N/A |  |
| input4: structureForComparison | 0 | 1 | N/A |  |
| output1: RawInteractionNetwork | 0 | 1 | N/A |  |
| **Merger step** | 0 | 1 | N/A | The original article does not mention this step, but once the two SMAP steps are apparent then the use of a merger step becomes obvious. |
| component | 0 | 1 | N/A |  |
| input1: RawInteractionNetwork1 | 0 | 1 | N/A |  |
| input2: RawInteractionNetwork2 | 0 | 1 | N/A |  |
| output1: mergedOutput | 0 | 1 | N/A |  |
| **AlignementResultMerger step** | 0 | 1 | N/A | The original article does not mention this step, but once the two SMAP steps are apparent then the use of a merger step for gathering the alignement results becomes obvious |
| component | 0 | 1 | N/A |  |
| input1: RawInteractionNetwork1 | 0 | 1 | N/A |  |
| input2: RawInteractionNetwork2 | 0 | 1 | N/A |  |
| output1: mergedOutput | 0 | 1 | N/A |  |

Table S2: Reproducibility scores for the FATCAT workflow

| **Workflow Fragment/Component** | **MINIMAL** | **NOVICE** | **AUTHOR** | **JUSTIFICATION** |
| --- | --- | --- | --- | --- |
| **GetSignificantResults step** | 0 | 0 | 1 | This component is an intermediate step to a)Process the files resultant from the previous step; b)Produce a list for FATCAT in the appropriate format and a list of significant results to compare afterwards. The script provided by the authors showed this step. Experts could infer this step from a note in the article: “identify pairs of similar binding sites“. |
| software component | 0 | 0 | 1 |  |
| input1: MergedOutput | 0 | 0 | 1 |  |
| input2: TBproteinInfo | 0 | 0 | 1 |  |
| output1: List | 0 | 0 | 1 |  |
| output2: SignificantResults | 0 | 0 | 1 |  |
| **FATCATURLchecker step** | 0 | 0 | 1 | This step was not part of the original method. It was added to address the decay of protein IDs, and check whether the protein IDs are available in the PDB or they had been superseded by new ones. An expert would know to add this step in order to handle the changes in IDs. |
| component | 0 | 0 | 1 |  |
| input1: list | 0 | 0 | 1 |  |
| output1: CheckedList | 0 | 0 | 1 |  |
| **FATCAT step** | 1 | N/A | N/A | “the first chain of each PDB file was aligned using **FATCAT”** |
| component | 1 | N/A | N/A |  |
| input1: checkedList | 0 | 1 | N/A | The original article mentions **“the first chain of each PDB file** was aligned using FATCAT”. However, without relevant expertise it is not clear how these chains of PDB files are obtained from earlier steps. |
| configuration parameter (not explicit in workflow) | 0 | 0 | 1 | The original article mentions “those pairs with a significant P-value of less than 0.05 were discarded”. It seemed to be a configuration parameter, but it turns out to be part of how FATCAT works. There is no need for a configuration parameter, but only experts would know from the FATCAT publications. |
| output:comparisonOutput | 1 | N/A | N/A | “FATCAT reports the statistical significance of the structural similarity, measured as a P-value.” |
| **RemoveSignificantPairsFATCAT step** | 0 | 1 | N/A | This step is not explicitly mentioned in the article. However, someone with some degree of expertise would see that the results of the SMAP comparison and the results of the FATCAT comparison need to be combined. That is what this step does. |
| component | 0 | 1 | N/A |  |
| input1: ComparisonOutput | 0 | 1 | N/A |  |
| input2: SignificantResults | 0 | 1 | N/A |  |
| output1:negativeData | 0 | 1 | N/A |  |
| output2:significantResultsFiltered | 0 | 1 | N/A |  |

Table S3: Reproducibility scores for the docking workflow

| **Workflow Fragment/Component** | **MINIMAL** | **NOVICE** | **AUTHOR** | **QUOTE** |
| --- | --- | --- | --- | --- |
| CreateClipFiles step | 0 | 1 | N/A | “The aligned drug molecule was used as the clip file with a default search space of 10Å3” |
| software component | 0 | 0 | 1 | This step had to be written for the new tool (AutodockVina). Advanced expertise is required to write software for this step. |
| input1: SMAPAlignmentFolder | 0 | 1 | N/A | “Since SMAP had aligned the drug binding site with the M.tb protein binding site, the aligned coordinates of the drug molecule were used to define the search space for docking that drug into the M.tb protein.” The text indicates that the SMAP software gives this result, but it does not return this result explicitly. It takes some investigation into the SMAP software to see that these results are placed in a special folder by SMAP. |
| output1: ClipFiles | 0 | 1 | N/A | Advanced expertise is required to write software that generates the output needed. |
| CreateIdealLigandsImpl step | 0 | 0 | 1 | This step is not in the paper, and by looking at the Autodock Vina software it could not be inferred without some expertise. The authors had written scripts for this step. |
| component | 0 | 0 | 1 |  |
| input1: clipFiles | 0 | 0 | 1 |  |
| output1: idealLigandFiles | 0 | 0 | 1 |  |
| IdealLigandCheckerImpl step | 0 | 0 | 1 | This step had to be added in order to remove some input ligands that were not recognized by Autodock Vina. This step was not used in the original method because a different tool (eHits) was used. |
| step | 0 | 0 | 1 |  |
| component | 0 | 0 | 1 |  |
| input1: idealLigandFiles | 0 | 0 | 1 |  |
| output1: idealLigandsChecked | 0 | 0 | 1 |  |
| AutodockVina | 1 | N/A | N/A | “For those pairs of interest, molecular docking was used” |
| component | 0 | 0 | 1 | “eHiTS Lightning was selected due to its fast speed, relatively high accuracy and ease of automation for large-scale docking studies”.  The article mentioned proprietary software that was no longer accessible for this work. |
| input1: SMAPAlignementFolder | 1 | N/A | N/A | “Since SMAP had aligned the drug binding site with the M.tb protein binding site, the aligned coordinates of the drug molecule were used to define the search space for docking that drug into the M.tb protein.” |
| input2: IdealLigandsChecked | 0 | 0 | 1 | This input was not mentioned in the paper, and the software and scripts provided by the authors did not need this input. Advanced expertise is required to determine how to create this new input for Autodock Vina. |
| input3: SignificantResultsFiltered | 0 | 1 | N/A | These inputs were not specified in the original paper, but the software describes what inputs are needed. |
| input4: SolvedStructureFiles | 0 | 1 | N/A |  |
| output1: DockingResults | 1 | N/A | N/A | “For those pairs of interest, molecular docking was used to predict the binding pose and affinity of the drug molecule to the M.tb protein” |

Table S4: Top rated highly connected drugs (original results)

| **Drug Name** | **Connections** | **Connections-solvedStructures** | **M.tb proteins with Solved structures** |
| --- | --- | --- | --- |
| Alitretinoin | 98 | 14 | aroG, bioD, bpoC, cyp125, embr, glbN, inhA, IppX, nusA, pknE, prcA/prcB, purN, Rv1264, Rv3676 |
| Levothyroxine | 63 | 14 | argB,bioD,blal,ethR,glbO,kasB,IrpA,nusA,pcrA,Rv1264,Rv3676,secA1,thyX |
| Methotrexate | 48 | 10 | argB,aroF,cmaA2,cyp121,cyp51,lpd,mmaA4,panC,Rv3676,TB31.7 |
| Estradiol | 38 | 10 | argB,bphD,cyp121,cysM,InhA,mscL,pknB,Wv1264,sigC |
| Rifampin | 34 | 6 | inA,lpdA,lppX,mscL,prpB,Rv3676 |
| 4-Hydroxytamoxifen | 33 | 10 | argB,cysM,inhA,katG,LppX,pknB,pknE,Wv1264,Rv1941,Rv3676 |
| Amantadine | 32 | 0 | homology models only |
| Raloxifene | 28 | 10 | deoD,inhA,mbtK,pknB,pknE,prcA/prcB,Rv1264,Rv3676,secA1msigC |
| Propofol | 24 | 3 | clpP,glbN,InhA |
| Indinavir | 23 | 2 | InhA, IpdA |
| Ritonavir | 22 | 7 | accD5,arok,fabH,IpdA,panC,serA1,TB31.7 |
| Darunavir | 22 | 5 | cyp124,devB,InhA,IpdA,panC |
| Lopinavir | 22 | 4 | IpdA,nrdB,pknG,tpiA |
| Penicillamine | 20 | 5 | groEL,inhA,nusA,Tv1264,Rv3676 |
| Nelfinavir | 20 | 3 | fabH,pknG,serA1 |

**Table S5: Top rated highly connected drugs, results of the workflow. The entries found in the original results have been highlighted in order to show the commonalities between both.**

| **Drug Name** | **Connections** | **Connection-SolvedStructures** | **M.tb proteins with Solved structures** |
| --- | --- | --- | --- |
| **Tretinoin** | 257 | 46 | **aroG, bioD, cyp125, embR, glbN, inhA, lppX, nusA, pknE, purN, Rv1264 ,Rv3676,** Rv1155, mscL,thyX,gmk,glnA1,Rv0802c,Rv0948c,ptbB,uppS,blaI,ethR,sigC,bphD,pepD,Rv3361c,pth,argB,cyp51,Rv2991,lpd,lppx,suhB,glmU,cysK1,PPE41, PE25,nirA,coaA,pcA,cmaA1,embR,pknB,fprA,mog,Rv3472,Rv0760c, |
| **Levothyroxine** | 173 | 36 | **argR,bioD,blaI,ethR,glbN,glbO,kasB,lrpA,nusA,prrA,Rv1264,Rv3676,secA1,thyX,**icL,glnA1,bpoC,lppX,trpD,leuA,coaA,sodA,trxB2,ephB,pknE,aroK,gpgS,recA,ruvA,clpP,inhA,argB, PPE41, PE25, Rv2714, pcaA, mmaA4 |
| **Methotrexate** | 156 | 32 | **argB,aroF,cyp121,cyp51,lpd,mmaA4,panC,Rv3676,TB31.7,**Rv0223c,lipJechA6,Rv1264,lppX,secA1,cysK1,ephG,devS,Rv2714,blaI,pcaA,ethR,Rv0948c, aroG,sigC,glbN,bph,DclpP,inhA,mshD, lipJ, bphD, clpP, echA6, mmaA2, nirA, cmaA1 |
| **4-Hydroxytamoxifen** | 115 | 25 | **cysM,inhA,pknB,pknE,Rv1264,Rv1941,Rv3676,**cyp130,lppX,gpm1,ligA,folC,bioD,Rv0948c,aroG,groEL,mbtK,mbtI,cyp121,Rv0760c,recA,panC,clpP,adk, nirA,pcaA |
| **Estradiol** | 98 | 20 | **argB,bphD,cyp121,cysM,inhA,mscL,pknB,Rv1264,Rv3676,sigC,**TB31.7,lppX,coaA,cmaA1,glbN,Rv0760c,panC,clpP, ethR,pcA |
| **Amantadine** | 79 | 1 | fabG1 |
| **Rifampin** | 78 | 13 | **inhA,lpdA,lppX,mscL,ptbB,Rv3676,**mmaA4,bphD,Rv1264,thyX,ruvA,mmA2,ethR |
| **Raloxifene** | 75 | 18 | **inhA,mbtK,pknB,pknE,Rv1264,Rv3676,secA1,sigC,**TB31.7,cyp130,aroG,trpD,coaA,cysM,Rv0813c,dfrA,Rv0760c,nirA |
| **Propofol** | 54 | 5 | **clpP,glbN,inhA,**pth,ethR, |
| **Indinavir** | 51 | 14 | **lpdA,inhA,**pknD,lipJ,fabH,Rv1941,Rv3361c,Rv1264,Rv0802c,serA1,accD5,panC,Rv3676,lppX |
| **Penicillamine** | 44 | 10 | **groEL,inhA,nusA,Rv1264,Rv3676,**lppX,secA1,glmU,glbN, mmaA4 |
| Daunorubicin | 44 | 12 | mmaA4,Rv1264,thyX,lppX,secA1,serA1,Rv3529c,coaA,ptbB,cyp124,ethR,inhA, |
| Triclosan | 42 | 5 | pepD,Rv1264,thyX,ethR,trxB2 |
| **Darunavir** | 40 | 15 | **cyp125,devB,inhA,lpdA,panC**,pknD,pepD,fabH,Rv1941,ppp,ftsZ,hrp1,argR,Rv3676,adk, |
| Desoxycorticosterone | 39 | 12 | mmaA4,Rv0813c,Rv1264,bpoC,thyX,lppX,cysK1,nusA,Rv0760c,sahH,pcaA,embR, |
| Diethylstilbestrol | 39 | 7 | folP2,Rv1264,pcaA,Rv3676,aroK,mshB,inhA, |
| Amprenavir | 38 | 14 | pknD,pepD,fabH,Rv3361c,ftsZ,lppX,hrp1,argR,panC,Rv3676,folC,nrdB,trxB2,adk, |
| Tadalafil | 36 | 7 | nirA,coaA,Rv3676,lppX,secA1,mshB,inhA, |
| Pemetrexed | 35 | 17 | icl,cyp130,Rv1264,lppX,secA1,serA1,mmaA2,ephG,devS,Rv3676,pknG,cmaA1,glbN,rph,cyp121,cyp124,inhA, |
| **Lopinavir** | 35 | 10 | pknD,cyp51,pepD,fabH,ppnK,hrp1,argR,Rv3676,trpB,inhA, |
| Saquinavir | 34 | 13 | pknD,fabH,Rv1941,cyp130,devB,cyp121,serA1,ephG,hrp1,argR,accD5,adk,inhA, |
| Indomethacin | 34 | 8 | cyp51,cmaA2,gpm1,coaA,Rv3676,ethR,glbN, inhA |
| Bepridil | 33 | 14 | cyp51,Rv1264,bpoC,pknE,secA1,cyp121,mmaA2,Rv3676,ethR,nrdB,ruvA,kasB,sigC,inhA, |
| Testosterone | 32 | 14 | bphD,Rv1264,groEL,bpoC,ftsZ,glbO,secA1,leuA,Rv3676,ethR,clpP,bioD,lpdA,kasB, |
| Bexarotene | 32 | 10 | Rv1264,mscL,pknE,lppX,glnA1,mshB,uppS,pcaA,Rv3676,inhA, |
| Imatinib | 32 | 12 | mmaA4,groEL,lppX,secA1,trpD,coaA,lrpA,Rv3676,cmaA1,ald,dapB,inhA, |
| **Ritonavir** | 32 | 10 | **fabH,panC,lpdA,TB31.7,**lipJ,Rv0802c,prcA, prcB,fprA,inhA, lppX |
| **Nelfinavir** | 28 | 9 | **fabH,pknG,**pknD,pepD,hrp1,argR,Rv3676,desA2,inhA, |
